# RANKL/RANK signaling recruits Tregs via the CCL20–CCR6 pathway and promotes stemness and metastasis in colorectal cancer

**DOI:** 10.1038/s41419-024-06806-3

**Published:** 2024-06-20

**Authors:** Jing Ouyang, Shuang Hu, Qingqing Zhu, Chenxin Li, Tingting Kang, Wenlin Xie, Yun Wang, Yan Li, Yingsi Lu, Junhua Qi, Ming Xia, Jinrun Chen, Yingqian Yang, Yazhou Sun, Tianshun Gao, Liping Ye, Qian Liang, Yihang Pan, Chengming Zhu

**Affiliations:** 1 Scientific Research Center, The Seventh Affiliated Hospital, Sun Yat-sen University, Shenzhen, 518107 China; 2 Guangdong Provincial Key Laboratory of Digestive Cancer Research, Shenzhen, 518107 Guangdong China; 3 Department of Oncology, The First Affiliated Hospital of Zhengzhou University, Zhengzhou, 450052 China; 4 Pathological Diagnostic Center, The Seventh Affiliated Hospital, Sun Yat-sen University, Shenzhen, 518107 China; 5 Department of Clinical Medical Laboratory, The Seventh Affiliated Hospital, Sun Yat-sen University, Shenzhen, 518107 China; 6 Clinical Big Data Research Center, The Seventh Affiliated Hospital, Sun Yat-sen University, Shenzhen, 518107 China; 7 Department of Spine Surgery, The First Affiliated Hospital of Shenzhen University, The Shenzhen Second People’s Hospital, Shenzhen, China

**Keywords:** Prognostic markers, Chemokines

## Abstract

TNF receptor superfamily member 11a (TNFRSF11a, RANK) and its ligand TNF superfamily member 11 (TNFRSF11, RANKL) are overexpressed in many malignancies. However, the clinical importance of RANKL/RANK in colorectal cancer (CRC) is mainly unknown. We examined CRC samples and found that RANKL/RANK was elevated in CRC tissues compared with nearby normal tissues. A higher RANKL/RANK expression was associated with a worse survival rate. Furthermore, RANKL was mostly produced by regulatory T cells (Tregs), which were able to promote CRC advancement. Overexpression of RANK or addition of RANKL significantly increased the stemness and migration of CRC cells. Furthermore, RANKL/RANK signaling stimulated C-C motif chemokine ligand 20 (CCL20) production by CRC cells, leading to Treg recruitment and boosting tumor stemness and malignant progression. This recruitment process was accomplished by CCL20–CCR6 interaction, demonstrating a connection between CRC cells and immune cells. These findings suggest an important role of RANKL/RANK in CRC progression, offering a potential target for CRC prevention and therapy.

## Background

Colorectal cancer (CRC) is one of the most prevalent forms of cancer on a global scale [1]. Despite considerable advancements in treatment techniques, the responses of patients with CRC continue to be suboptimal. The prognosis of metastatic CRC (mCRC) continues to be unfavorable [2] as the disease is largely resistant to treatments. Hence, it is imperative from a clinical standpoint to investigate the underlying process and identify novel targets for the prevention of CRC.

In general, the tumor microenvironment (TME) interacts with cancer cells to decide the final fate of tumor development and migration [3]. The role of regulatory T cells (Tregs) in CRC progression is still debatable [4, 5]. Currently, researchers are primarily interested in the role of Tregs in promoting or suppressing cancer by controlling immune response; however, the direct interaction between Tregs and CRC cells has received less attention [6, 7].

There is emerging evidence suggesting that cancer stem cell (CSC) dysregulation plays an essential role in CRC growth and metastasis [8]. CSCs are a type of tumor cells with stem cell features that can self-renew and differentiate, leading to tumor development, therapy resistance, metastasis, and recurrence [9]. CSCs are typically formed as a result of numerous genetic alterations. Many unique genetic changes have been discovered as activating proto-oncogenes or inactivating tumor suppressor genes. Oncogenes and tumor suppressor genes are frequently assigned separate functions in tumor progression. However, the link between these genes and tumor stemness and advancement is still unknown.

All tumor necrosis factor (TNF) superfamily members exert a proinflammatory effect, which is mediated in part by the transcription factor NF-κB [10]. TNF receptor superfamily member 11a (RANK) and its ligand TNF superfamily member 11 (TNFRSF11, RANKL) have also been linked to cancer. Namely, RANKL/RANK signaling has been shown to cause the migration of human epithelial cancer cells and melanoma cells [11] and has also been examined in mammary epithelial cells and prostate epithelial cells [12]. Previous research has shown that RANKL/RANK signaling can promote CRC metastasis [13]. However, the specific mechanism by which RANKL/RANK signaling maintains CRC stemness and promotes metastasis remains unknown.

RANKL/RANK signaling promotes tumor growth by influencing multiple downstream pathways, including tumor metabolism, treatment resistance, and tumor immunity. RANKL has been linked to an increase in tumor-infiltrating lymphocytes and cancer metastasis [14]. RANKL/RANK signaling is also important for the formation of Tregs [15]. RANKL can be produced by CD4^+^ CD25^+^ T cells [16], and the majority of T cells that produce RANKL [17] also express the forkhead box P3 (FOXP3), a transcription factor produced by Tregs. Furthermore, soluble RANKL released into the TME can recruit Tregs via RANKL/RANK signaling [18]. In breast tumors, lack of RANK signaling promotes lymphocyte and CD8^+^ T-cell infiltration while decreasing macrophage and neutrophil infiltration [19]. However, the specific interaction network involving RANKL/RANK, immune cells, and tumor cells has not yet been elucidated.

In this study, we discovered that RANKL was mostly released by Tregs and that RANKL/RANK signaling was able to increase the malignant development of CRC by boosting tumor stemness. Further analysis indicated that this process was accomplished via activating the NF-κB pathway’s phosphorylation of P65. We also discovered that RANKL/RANK upregulated C-C motif chemokine ligand 20 (CCL20) production via the NF-κB pathway and recruited Tregs via the CCL20–CCR6 axis, thereby producing a “vicious cycle” in the TME. Thus, RANKL/RANK suppression may be evaluated as a potential new target for the therapy of CRC metastases.

## Materials and methods

### Clinical samples

Sun Yat-Sen University’s Seventh Affiliated Hospital and First Affiliated Hospital provided clinical samples. Tables S1 and S6 contain patient information. The stages were determined in accordance with UICC-TNM grading, and all samples were pathologically examined. All patients provided written informed permission in accordance with the Hospital’s Institutional Review Board guidelines. The use of clinical samples was approved by the Ethics Committee of Sun Yat-sen University.

### Antibodies and reagents

Primary antibodies used in this study included anti-RANK (ab13918), anti-FOXP3 (ab20034), anti-CCL20 (ab106009), anti-RANKL (ab9957), and anti-CD4 (ab133616) from Abcam, UK; anti-P65 (#4764), anti-p-P65 (Ser536; #3033), and anti-FLAG (#14793) and anti-IgG isotype control (#3900 S) from Cell Signaling Technology, CST, USA; anti-GAPDH (60004-1-lg), anti-CD44 (15675-1-AP), anti-CD133 (66666-1-Ig), and anti-CCR6 (66801-1-Ig) from Proteintech, Wuhan, China; T-bet (SC21749), GATA-3 (SC269), and RORγ (SC365476) from Santa Cruz, USA; and anti-CD56 (GB112671) from Servicebio, Wuhan, China. The reagents included QNZ from MCE; and RANKL, EGF, and bFGF from PeproTech.

### Cell lines and cell culture

All cells were obtained from the American Type Culture Collection (ATCC). They were authenticated using short tandem repeat (STR) profiling and were mycoplasma-free. The cells were cultured in RPMI 1640 supplemented with 10% fetal bovine serum (FBS) at 37 °C with 5% CO_2_. RANKL at 100 ng/mL and QNZ at 50 nm were introduced. T Cell Expansion Medium (STEMCELL) was used to culture human peripheral blood mononuclear cells (PBMCs) in accordance with the manufacturer’s recommendations.

### Establishment of stable cell lines and transient transfection

Plasmids were introduced into cells using Liposomal Transfection Reagent (YiSheng, Shanghai, China) in accordance with the directions for transient transfection; for stable transfection, 293 T cells were transfected with plasmids and polyethyleneimine (Polysciences, USA). Polybrene (Sigma, USA) was utilized to infect cells with virus particles. Puromycin (Sigma) was used to test plasmids for puro-resistance. Transheep (Shanghai, China) supplied all of the plasmids. Supplementary Tables S2 and S3 present siRNA and shRNA sequences.

### Real-time quantitative PCR (QRT-PCR)

RNA was extracted with AG RNAex Pro Reagent. QRT-PCR was performed using 5X Evo M-MLV RT Master Mix and SYBR^®^ Green Premix (AG). GAPDH was used as an internal reference, and specific primer sequences are shown in Table S4.

### Chromatin immuno-precipitation (ChIP) assay

The ChIP assay was performed using a ChIP assay Kit (Beyotime, Shanghai, China). Before adding cell lysates, magnetic beads were combined with anti-P65 and anti-rabbit IgG. Then, DNAs were purified. QRT-PCR was used for further analyses.

### Enzyme-linked immunosorbent assay (ELISA)

The content of CCL20 and RANKL in the supernatants was determined using ELISA kits (4 A Biotech, Beijing, China).

### Western blotting (WB), immunohistochemistry (IHC), and immunofluorescence (IF)

RIPA Lysis Buffer (PC101, Yamei, Shanghai, China) was used for protein extraction. SDS-PAGE was used to separate protein samples, which were then transferred to a nitrocellulose membrane (Merck Millipore, Germany). The membrane was treated with the primary antibody at 4 °C overnight. Secondary antibodies were IRDye 800CW Goat anti-IgG (LI-COR, USA), which were then seen using the ChemiDocTM MP Imaging System (BIO-RAD, USA) [13]. Images of uncropped Western blots are provided as supplementary information file.

Cells were fixed with 4% paraformaldehyde and stained with primary antibody on a sliding plate for IF staining. A fluorescent secondary antibody was added and incubated at room temperature. Finally, the nuclei were labeled with DAPI. Tissue IF staining was performed using the TSA PLus Kit (Servicebio). A secondary antibody was created using a DAB staining solution kit (Gene Tech, Shanghai, China).

For IHC, the slices were treated with primary antibodies at 4 °C overnight [13]. A second antibody was incubated on the second day. For staining, a DAB staining solution kit was utilized. A Leica DM4B microscope was used to examine the sections. We calculated the IHC scores as previously reported [20].

### Flow cytometry and cell sorting

PBMCs were isolated from blood samples using Ficoll (TBD)-gradient centrifugation. A Tumor Dissociation Kit (Miltenyi Biotec, Germany) was used for extracting lymph node cells. EasySep^TM^ Human T Cell Enrichment Kit (STEMCELL, Canada) was used to separate CD3^+^ T cells from PBMCs.

Human PBMCs and lymph node cells were stained for surface analysis with CD4-AF700 (#344622), CD25-PE/Cy7 (#302612), CCR6/PE (#353410), and RANKL-APC (#347508), and Treg intracellular analysis with FOXP3-BV421(#320124). All flow cytometry antibodies were purchased from Biolegend, USA. Before staining for FOXP3, the True-Nuclear^TM^ Transcription Factor Buffer Set (Biolegend) was used.

### Migration and chemotaxis assay

A total of 1 × 10^5^ cells were inoculated into the upper chamber (8 μm, Corning, USA). The medium was supplemented with 10% FBS as a chemotactic agent in the inferior cavity. After 48 h, the cells on the lower membrane were stained. The stained cells were randomly imaged using a microscope in five different fields.

Induced T cells were migrated and placed in the upper chamber of 24-well Transwell plates with a 3.0-μm polycarbonate membrane (SPL, Korea) (1 × 10^6^ cells/well). DLD1 WT/RK cells were grown in ImmunoCult^TM^-XF T Cell Expansion Medium (STEMCELL), and the supernatant was collected and deposited in the bottom chamber with or without anti-CCL20. Migration was permitted to continue for 6 h [21]. Flow cytometry was used to calculate the fraction of CD4^+^ FOXP3^+^ cells in the bottom chamber.

### Tumor sphere formation assays

Cells were seeded into 24-well ultralow attachment plates (5000 cells/well) and resuspended in the stem cell–conditioned medium containing DMEM-F12 (Bio-channel, Nanjing, China), 2% B-27 Supplement (PeproTech), 10 ng/mL basic fibroblast growth factor (bFGF, PeproTech), 20 ng/mL epidermal growth factor (EGF, PeproTech), and 5 µg/mL insulin (Beyotime), at 37 °C with 5% CO_2_ and saturated humidity for 12–14 days. When spheroid diameters reached 50 μm, culture suspensions were passed every 7 days. Photographs were taken, and the number of cell spheres was tallied.

### Subcutaneous xenograft implantation models and in vivo limiting dilution assay

The animal research was carried out with the agreement of the Sun Yat-sen University’s Animal Experiment Ethics Committee. Guangdong Yaokang Biotechnology Co., Ltd., provided female NOD-Scid mice (4–6 weeks). No animals were excluded from the analysis.

For subcutaneous xenograft assays, mice were randomly divided into two groups. DLD1 cells or DLD1 RK cells (2 × 10^7^ cells per mouse) were subcutaneously injected into NOD-Scid mice (*n* = 5). After 3 weeks, the mice were sacrificed, and xenograft tumors were harvested for histological study. The tumor volume was calculated as follows: Volume (mm^3^) = width^2^ (mm^2^) × length (mm) × 0.4 [22].

For limiting dilution assays, mice were randomly divided into six groups. DLD1 cells or DLD1 RK cells were implanted at the gradient of 1 × 10^6^, 1 × 10^7^, and 2 × 10^7^ cells per NOD-Scid mouse (*n* = 5 per group). The incidence of tumors (DLD1 WT/RK cells) was analyzed. The frequency of tumor stem cells was calculated by a method described on the ELDA website (Extreme Limiting Dilution Analysis, http://bioinf.wehi.edu.au/software/elda/).

### Statistical analysis

Sample size descriptions are detailed in figure legends or main text. No data points were excluded from this study. All in vitro assays were performed in biological triplicates and error bars on bar plots indicate mean + SD unless stated otherwise. Within each group, there is an estimate of variation, and the variance between groups is similar. SPSS and GraphPad Prism were used for statistical analysis. ImageJ was used to calculate protein levels. GAPDH was used to standardize expression levels. CytExpert and FlowJo were used to evaluate flow cytometry data. Differences between groups were tested using the student’s t-test or one-way ANOVA. All the statistical tests were justified for every figure and the data met the assumptions of the tests. The χ^2^ test was performed to investigate the association between various biomarkers in CRC tissue slices. The survival curve was created using the Kaplan–Meier method and the log-rank test. Statistical significance was established at two-sided *P* < 0.05. Pearson’s correlation coefficient and *p*-values are displayed in the graph. The investigators were not blinded to data allocation during experiments and outcome assessment.

### Public clinical datasets

Correlation analysis between gene expression in tissues of CRC patients was computed from Gene Expression Profiling Interactive Analysis (GEPIA, http://gepia.cancer-pku.cn/). ChIP-sequencing datasets were collected from the Cistrome Data Browser (http://cistrome.org/db/#/). Single-cell data were derived from a previously published article [23].

## Results

### RANKL/RANK expression is linked to the advancement of CRC malignancy

**Fig. 1:**
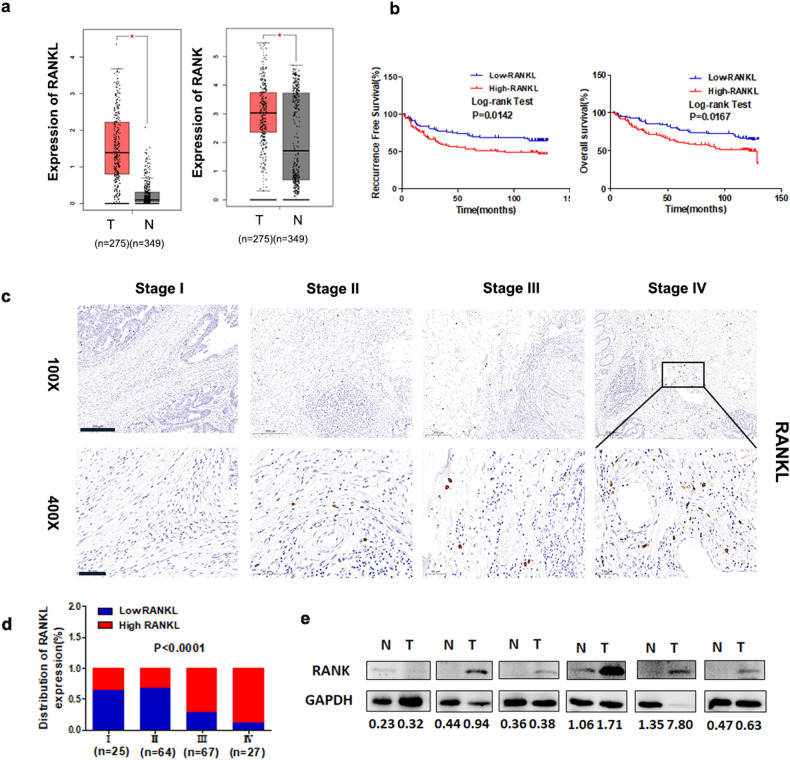
RANKL/RANK expression is associated with the malignant progression of CRC. **a** The mRNA levels of RANK and RANKL in CRC (*n* = 275) and normal colorectal (*n* = 349) tissues were determined by analyzing the GEPIA dataset. **b** Kaplan–Meier analysis of overall survival (OS) and recurrence-free survival (RFS) in CRC patients (*n* = 183). **c** RANKL immunohistochemistry staining in CRC tissues (stage I, stage II, stage III, and stage IV) (100x, 400x). **d** Percentage of RANKL expression in CRC tumors (*n* = 183) within individual TNM stage (*P* < 0.0001). **e** RANK protein expression was measured using WB in six matched tumor and normal tissue samples. Scales bars = 200 μm (100×), 100 μm (200×), and 50 μm (400×). *P < 0.05, **P < 0.01, ***P < 0.001.

We previously found that RANK expression was elevated in CRC patient samples and was linked to a poor prognosis [13]. We concentrated on RANKL in this investigation. In the TCGA dataset, RANKL was substantially elevated in 275 CRC tissues compared with 349 normal tissues (Fig. 1a). Furthermore, as demonstrated in Table S1, RANKL expression was associated with CRC clinical histology. Patients with increased RANKL protein expression had shorter overall survival (OS) and recurrence-free survival (RFS) among CRC patients having both OS and RFS data (Fig. 1b). IHC assays were utilized to compare RANKL protein expression between formalin-embedded tumor tissues from different TNM stages (Fig. 1c). The findings revealed that RANKL in CRC tumor tissues was significantly associated with TNM staging (*P* < 0.0001; Fig. 1d). WB demonstrated that RANK levels in CRC tumor tissues were higher than those in neighboring normal tissues (Fig. 1e), which was consistent with our previous findings. These findings indicated a high elevation of RANKL/RANK in CRC patients.

### RANKL is mainly generated by CD4^+^ CD25^+^ regulatory T cells in CRC

**Fig. 2:**
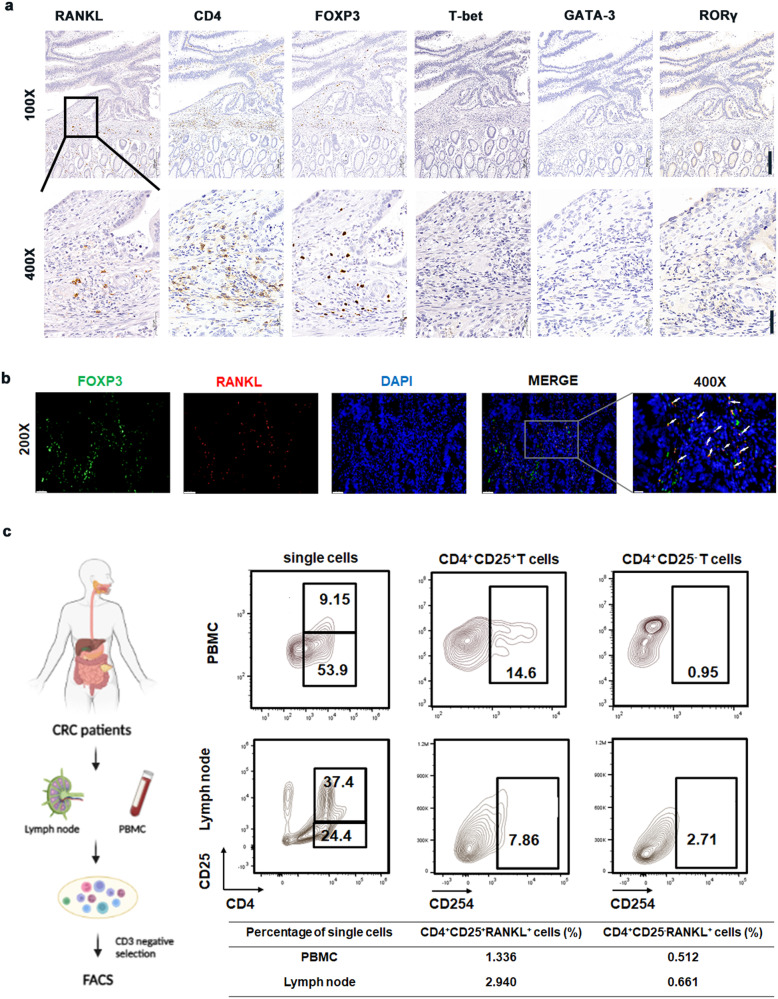
RANKL is mainly derived from CD4^+^ CD25^+^ regulatory T cells in CRC. **a** IHC co-stains RANKL with numerous immune cell markers (RORγ, GATA3, T-bet, FOXP3, CD4, RANKL) in CRC patients. (100×, 400×). **b** Colorectal tumor tissues subjected to immunofluorescence for FOXP3 (green), RANKL (red), and DAPI (blue). Two representative micrographs are shown (200×, 400×). **c** The expression of RANKL (CD254) in CD4^+^ CD25^+^ cells and CD4^+^ CD25^-^ cells were detected by flow cytometry in PBMC and Lymph nodes of CRC patients (The illustration created with BioRender.com). Scales bars = 200 μm (100×), 100 μm (200×), and 50 μm (400×).

We then attempted to locate the source of RANKL in CRC. Our previous research results [13] showed that RANKL colocalized with CD4^+^ T cells more than with other immune cells (CD8^+^ T cells, B cells, macrophages) in CRC tissues. Single-cell sequencing data (Fig. S1a) showed that RANKL was primarily produced by T cells in breast cancer, specifically CD4 + T cells [23], which was similar to the findings from Wei Tan et al [16]. We also found by ELISA that T cells of CRC patients were able to secrete RANKL (s-RANKL) (Fig. S1b). We used IHC (NK cells: CD56) of the CRC samples to determine the subtype of T cells RANKL primarily originated from (Fig. S1c). We discovered that RANKL expression was substantially overlapping with CD4 expression (Fig. 2a), implying that RANKL was produced by CD4^+^ T cells. RANKL and FOXP3 were also colocalized in CRC tissues (Fig. 2b). Flow cytometric analysis of the peripheral blood and lymph node of CRC patients revealed that membrane RANKL (m-RANKL) was primarily expressed in CD4^+^ CD25^+^ T cells compared with CD4^+^ CD25^−^ T cells (Fig. 2c). We concluded that Tregs expressed RANKL.

### RANKL/RANK signaling enhances CRC cell stemness

**Fig. 3:**
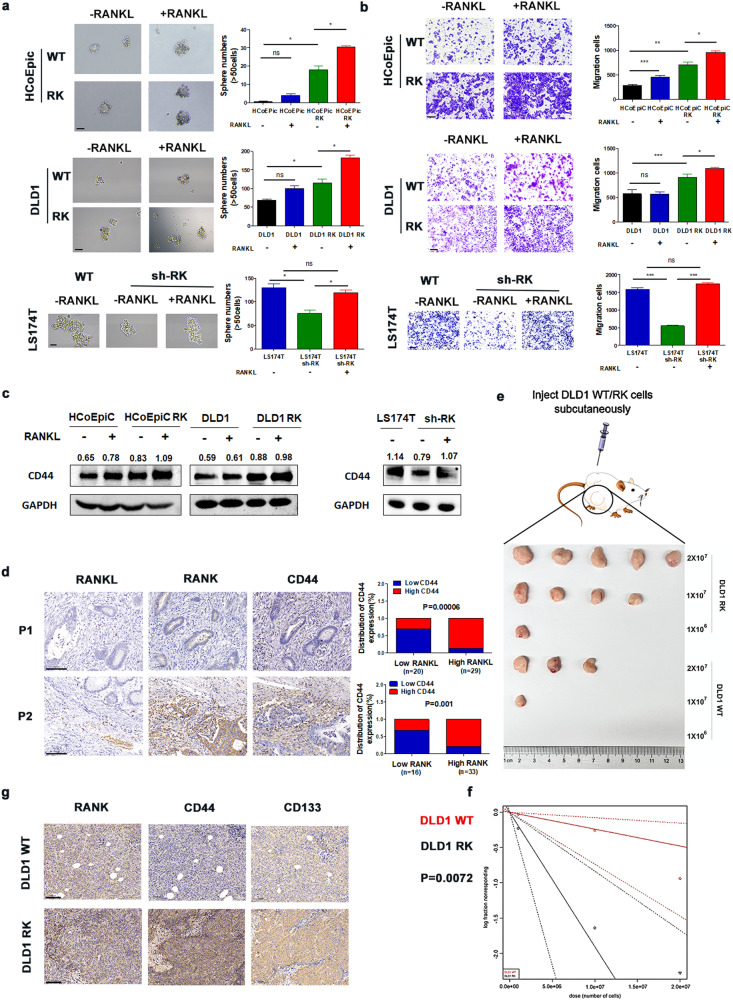
RANKL/RANK signaling promotes the stemness of CRC cells. **a** RANK overexpression or knockdown influenced the sphere formation (*n* = 3) and (**b**) migration (*n* = 5) of HCoEpiC, DLD1, and LS174T cells. And the addition of 100 ng/ml RANKL moderately increased the migration of HCoEpiC, DLD1, and LS174T cells. (Sphere formation: 400×, Migration: 200×). **c** WB of CD44 protein expression in RANK overexpression or knockdown cells, with 100 ng/ml RANKL added. **d** The relationship between the expression of RANKL, RANK, and CD44 in CRC tissues was detected by IHC (200×; P1: low expression, P2: high expression). (**e**) Tumor-initiating cell frequency was tested by in vivo limiting dilution assay in NOD/Scid mice (*n* = 5). **f** A graph of the log-fraction for the limiting dilution assay. DLD1 RK is represented by black circles, while control is represented by red circles. The dashed lines represent the confidence interval at 95%. **g** NOD/Scid mice with the specified subcutaneous tumors were stained with IHC to detect the expression of RANK, CD44, and CD133. (200×). Scales bars = 200 μm (100×), 100 μm (200×), and 50 μm (400×). *P < 0.05, **P < 0.01, ***P < 0.001, ns no significance.

To investigate whether RANKL/RANK may influence CRC stemness and metastasis, we evaluated TCGA CRC data and discovered that RANK/RANKL expression was positively associated with stemness-related genes (Fig. S2a). We also measured the RANK mRNA levels in other CRC cell lines (Fig. S2b); we chose HCoEpiC (normal intestinal epithelial cell line), DLD1, and Caco2 to create overexpressed cells, and LS174T to create knockdown cells. WB verified RANK-overexpressing cells (HCoEpiC RK, DLD1 RK, Caco2 RK) and RANK-knockdown cells (LS174T sh-RK) (Fig. S2c). Overexpression of RANK or addition of RANKL increased tumor sphere formation, whereas knocking down RANK was able to limit it (Fig. 3a, S2d). Our earlier study indicated that RANKL/RANK might accelerate CRC metastasis [13]. In the present investigation, we discovered that overexpressing RANK greatly boosted cell migration, whereas knocking down RANK inhibited migration (Fig. 3b, S2e). Furthermore, the inclusion of RANKL increased tumor sphere formation and CRC cell metastasis. Mechanistically, all CSC markers were upregulated in RANK-overexpressing cells (Fig. S3a), with CD44 and PROMI (CD133) being the most significant. The WB data verified this conclusion (Fig. 3c, S3b). Clinically, patients with elevated RANKL/RANK expression similarly had increased CD44 expression in CRC (Fig. 3d). The size and weight of the subcutaneous tumors generated by DLD1 RK cells were much larger than those generated by the control cells in vivo (Fig. S2f). To acquire a better understanding of the role of RANK in CSC stemness, we performed an in vivo limited dilution tumor transplantation investigation (Fig. 3e). Tumor-initiating cells were found in one out of every 5.248 × 10^6^ DLD1 RK cells and one out of every 4.174 × 10^7^ DLD1 cells, as illustrated in Fig. 3f. Notably, the frequency of tumor-initiating cells in DLD1 RK increased compared with the control. Table S5 shows the detailed numbers of cells planted and tumors formed. IHC labeling with CD44 and CD133 revealed that subcutaneous tumors generated by DLD1 RK cells had a higher stemness index than control cells (Fig. 3g). In conclusion, we found a substantial association between RANKL/RANK expression and tumor stemness.

### RANKL/RANK signaling increases the secretion of CCL20 by CRC cells

**Fig. 4:**
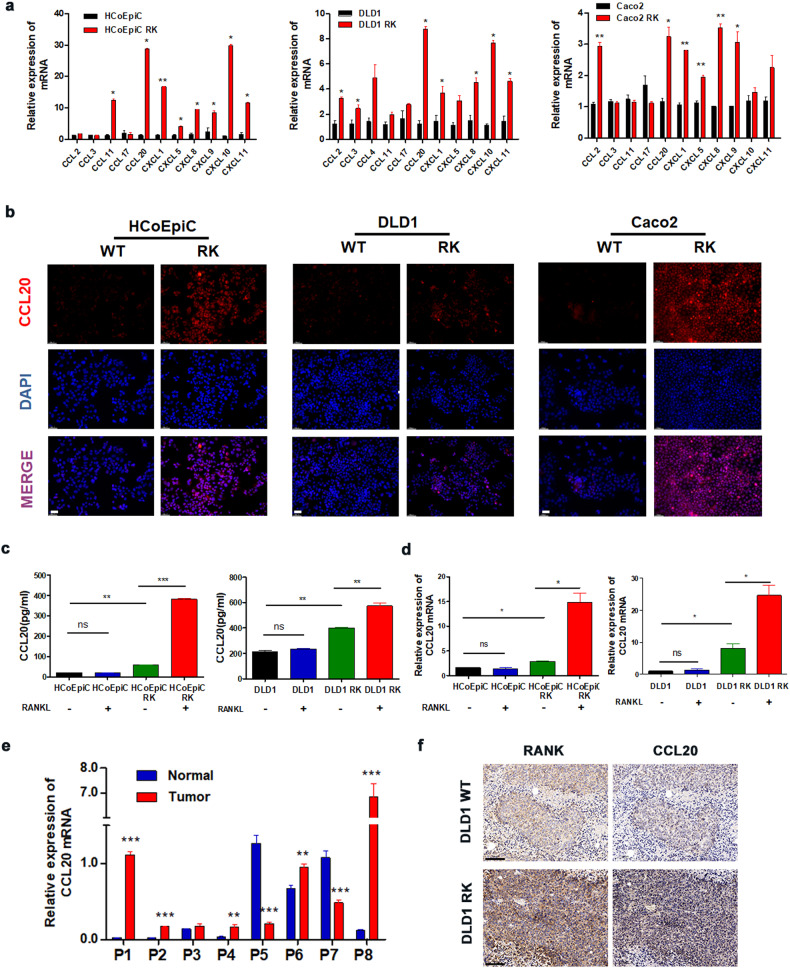
RANKL/RANK promotes the secretion of CCL20 by CRC cells. **a** QRT-PCR was used to analyze the relative expression of related chemokines in RANK-overexpressing cells (*n* = 3). **b** CCL20 protein expression via immunofluorescence in RANK overexpression cells. (200×). **c** ELISA (error bar indicates mean + SD of 2 technical replicates) and (**d**) QRT-PCR (*n* = 3) of CCL20 protein expressions in RANK overexpression cells, and the addition of 100 ng/ml RANKL. **e** CCL20 mRNA expressions in eight paired tumor and normal tissue samples. Expression levels were normalized with GAPDH. T human CRC tissues, N paired normal colorectal tissues (P1–8: eight different CRC patients). **f** IHC staining was used to detect the expression of RANK and CCL20 in indicated subcutaneous tumors of NOD/Scid mice. (200×). Scales bars = 200 μm (100×), 100 μm (200×), and 50 μm (400×). *P < 0.05, **P < 0.01, ***P < 0.001.

To identify critical immune-related factors for the poor prognosis in CRC patients with high RANK expression, we examined levels of chemokines. Most chemokine mRNA levels were elevated in RANK-overexpressing cells in vitro (Fig. 4a), with CCL20 being the most significant, which was similar to the findings from Liu et al [24]. TCGA CRC data showed that RANK/RANKL expression was strongly linked to CCL20 (Fig. S4a). Furthermore, we discovered that CCL20 mRNA levels in CRC cell lines were higher than those in normal cell lines (HCoEpiC) (Fig. S4b).

We performed IF staining and ELISA experiments to confirm our findings and discovered that CCL20 expression was upregulated in RANK-overexpressing cells (Fig. 4b, S4c). Furthermore, the addition of RANKL increased the levels of CCL20 protein and mRNA (Fig. 4c, d). Clinically, CCL20 mRNA expression levels in tumor tissue were higher than those in normal tissue in the majority of CRC patients (Fig. 4e). We also found that the CRC patients with high CCL20 expression had worse prognosis, as shown in Table S6. IHC labeling with CCL20 in vivo indicated a higher expression in the subcutaneous tumors generated by DLD1 RK cells than in control cells (Fig. 4f). By combining these findings, we concluded that RANKL/RANK signaling enhanced CCL20 production in CRC cells.

### Overexpression of RANK in CRC cells promotes recruitment of Tregs via the CCL20–CCR6 interaction

**Fig. 5:**
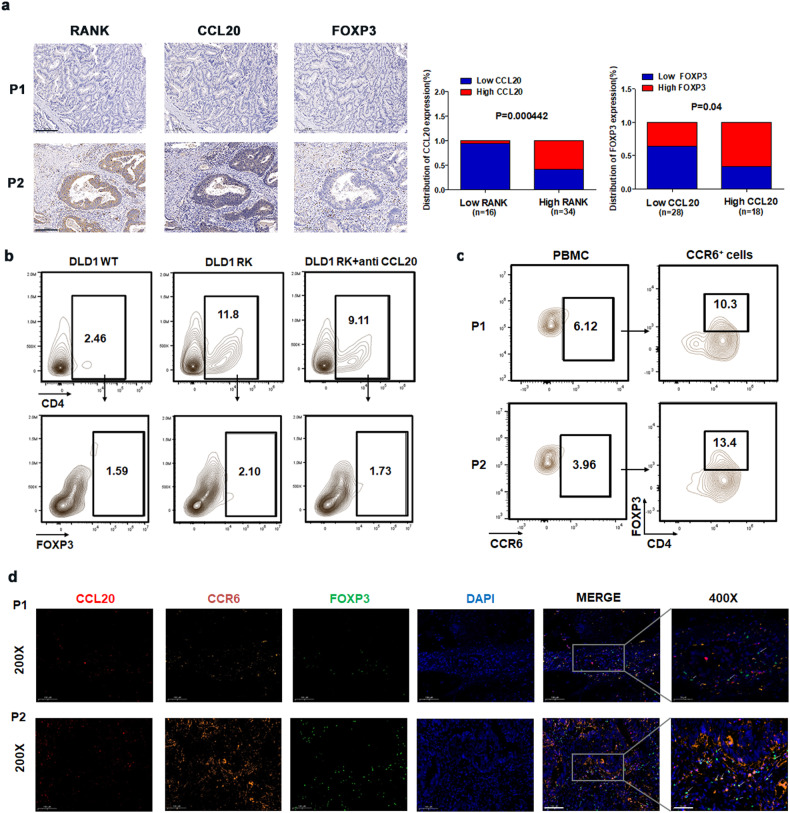
Overexpression of RANK in CRC cells promotes recruitment of Tregs via the CCL20-CCR6 interaction. **a** The relationship between the expression of RANK, CCL20, and FOXP3 in CRC tissues was detected by IHC (200 ×; P1: low expression, P2: high expression). **b** Migration of PBMC from CRC patients co-cultured with the supernatants of DLD1 cells or RANK-overexpressing DLD1 cells was analyzed by flow cytometry, and the addition of anti-CCL20 inhibitor. **c** The proportion of CD4^+^ FOXP3^+^ cells in CCR6^+^ cells was detected by flow cytometry in PBMC of CRC patients. (P1 and P2 are two different samples from CRC patients). **d** Colorectal tumor tissues subjected to three immunofluorescences for FOXP3 (green), CCL20 (red), CCR6 (orange), and DAPI (blue). Two representative micrographs are shown (200×,400×; P1: low expression, P2: high expression). Scales bars = 200 μm (100×), 100 μm (200×), and 50 μm (400×).

Numerous studies have demonstrated that the CCL20–CCR6 pathway can recruit Tregs [25, 26]. We found that tumor tissues with high expression of RANK or CCL20 were strongly associated with enhanced FOXP3 expression (Fig. 5a). To demonstrate that RANK can enhance CCL20 release and thereby attract Tregs, we performed an in vitro experiment in which DLD1 WT/RK cells were cocultured with PBMCs from CRC patients. DLD1 RK cell supernatants attracted substantially more Tregs (Fig. S5a). To confirm the critical role of CCL20, we added anti-CCL20, which inhibited the increased recruitment of Tregs by RANK-overexpressing DLD1 cells (Fig. 5b, S5). To confirm that RANKL/RANK signaling recruits Tregs via the CCL20–CCR6 pathway, we discovered that CCR6 and FOXP3 were colocalized in the blood (Fig. 5c) and tumor tissue (Fig. 5d) of CRC.

### RANKL/RANK signaling increases stemness and CCL20 production by CRC cells via NF-κB

**Fig. 6:**
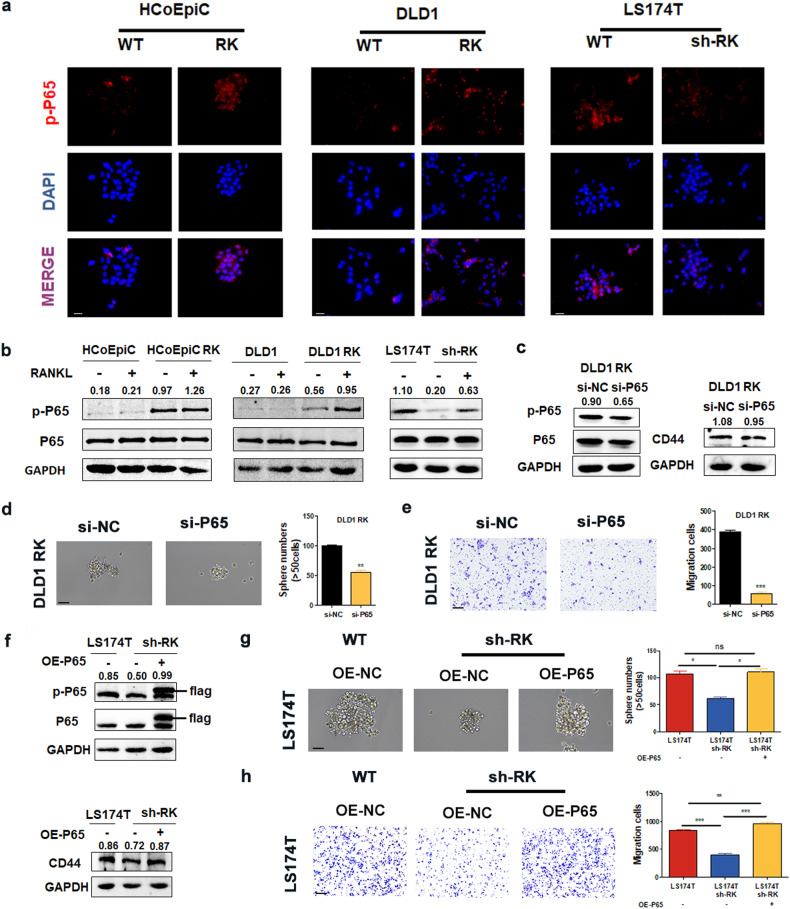
RANKL-RANK promotes the stemness via NF-κB signaling. **a** Immunofluorescence of p-P65 protein expressions in RANK overexpression or knockdown cells (400×). **b** WB of p-P65/P65 protein expressions in RANK overexpression or knockdown cells and the addition of 100 ng/ml RANKL. **c** Changes of p-P65/P65 protein and CD44 protein levels in DLD1 RK cells add P65 siRNA treatment. **d** RANK overexpression or P65 siRNA treatment influenced the sphere formation (*n* = 3) and (**e**) migration (*n* = 5) of DLD1 cells. (Sphere formation: 400×, Migration: 200×). **f** WB of p-P65/P65 and CD44 protein expressions in RANK knockdown cells and overexpressed P65. **g** RANK knockdown or overexpressed P65 influenced the sphere formation (*n* = 3) and (**h**) migration (*n* = 5) of LS174T cells. (Sphere formation: 400×, Migration: 200×). Scales bars = 200 μm (100×), 100 μm (200×), and 50 μm (400×). *P < 0.05, **P < 0.01, ***P < 0.001, ns no significance.

Since RANK is the receptor activator of NF-κB, we first determined whether RANKL/RANK–related promotion of stemness and CCL20 secretion was mediated by the activation of the NF-κB pathway. By WB and IF, we found that both overexpression of RANK and the addition of RANKL promoted the nuclear phosphorylation of P65 (Fig. 6a, b) and its entry into the nucleus (Fig. S6a). We investigated the role of the NF-κB pathway in the process of RANK-related promotion of stemness. An NF-κB pathway inhibitor (QNZ) significantly inhibited the phosphorylation of P65 (Fig. S6b), the expression of the CSC marker (Fig. S6c), and the ability of tumor sphere formation (Fig. S6d) and metastasis (Fig. S6e), as demonstrated by WB.

To further corroborate the crucial role of P65, we showed by WB (Fig. 6c) and QRT-PCR (Fig. S6f) that CD44 was also decreased after P65 was knocked down in CRC cells. In addition, tumor sphere formation and migration ability were weakened (Fig. 6d, e). By contrast, when we overexpressed P65 in LS174T sh-RK cells, we discovered that downregulation of CD44 could be rescued (Fig. 6f), along with the ability to form tumor spheres and migrate (Fig. 6g, h). IHC staining with p-P65 confirmed a higher expression in the subcutaneous tumors formed by DLD1 RK cells compared with control cells in vivo (Fig. S6g).

**Fig. 7:**
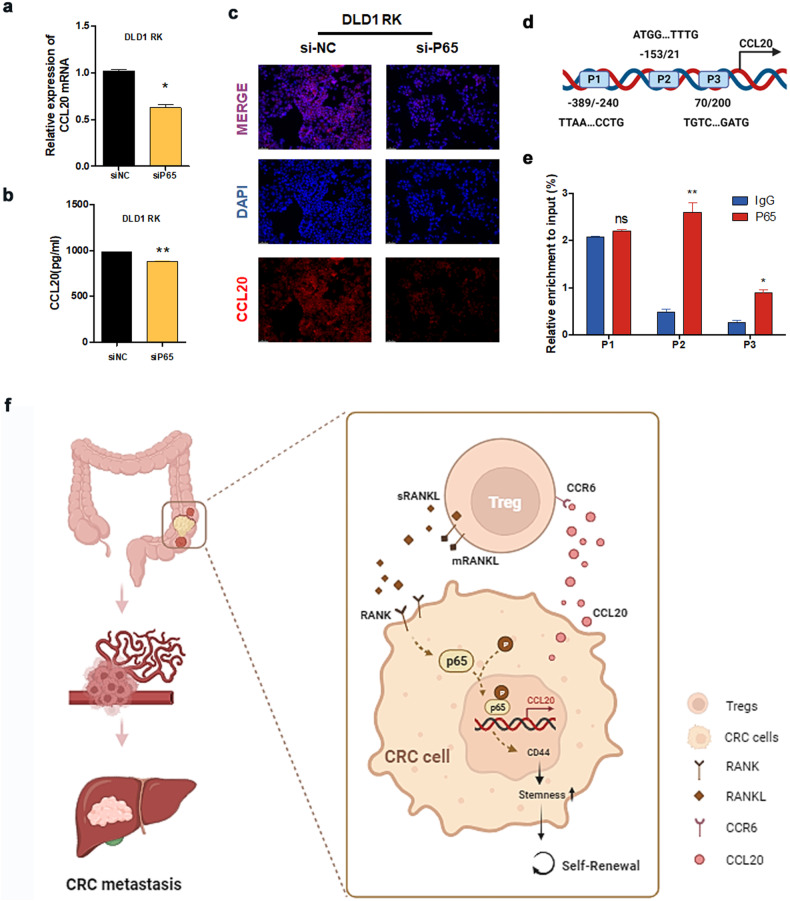
RANKL-RANK promotes the secretion of CCL20 by CRC cells via NF-κB signaling. **a** Changes of CCL20 mRNA (QRT-PCR, *n* = 3), (**b**, **c**) CCL20 protein (ELISA/immunofluorescence) in DLD1 RK cells add P65 siRNA treatment (200×). For ELISA, error bar indicates mean + SD of 2 technical replicates. **d** The schematic structures of P65 putative binding sites in the CCL20 promoter (Created with BioRender.com), and (**e**) ChIP analysis of P65 binding sites to the CCL20 promoter. Scales bars = 200 μm (100×), 100 μm (200×), and 50 μm (400×). *P < 0.05, **P < 0.01, ***P < 0.001, ns no significance. **f** The schematic illustration of working hypothesis (Created with BioRender.com). Tregs-derived RANKL activated RANK signaling pathway and upregulated the expression of downstream target genes CCL20, CD44 by activating the NF-κB pathway to recruit Tregs and maintain the CRC cancer stem cell characteristics and promotes metastasis.

The suppression of P65 significantly inhibited CCL20 expression (Fig. 7a–c). To confirm the relationship between P65 and CCL20 in CRC cells, we analyzed the Cistrome DB database and found P65-binding sites in the promoter of the CCL20 gene (Fig. 7d). After ChIP, three primer pairs corresponding to the predicted binding sites were used for QRT-PCR (Fig. 7e). The findings revealed that P65 interacted with the P2 ( − 153 to 21 bp) region of the CCL20 promoter. Collectively, our results indicate that P65 is a key target for RANK to promote CCL20 secretion in CRC.

## Discussion

RANKL/RANK signaling has been documented to closely correlate with metastasis of prostate cancer, breast cancer, liver cancer, and melanoma [11, 14, 27–29]. Our previous research [13] has demonstrated that RANK is associated with the progression of cancer in CRC. However, the RANKL/RANK signaling mechanism in CRC remains unknown. In this study, we discovered that the RANKL/RANK pathway promotes the secretion of chemokines that CRC cells can use to recruit Tregs, thereby influencing CRC stemness, metastasis, and tumorigenesis.

Although RANKL is best known for its distribution in bone [30], it has been reported on other cell types, such as immune cells, with aberrant expression found in certain tumor types [31]. Uncertain is the role of RANKL/RANK in tumor immunity. RANKL is derived from CD4^+^ FOXP3^+^ T cells in breast cancer and arthritis [16, 17]. In this study, we demonstrated (Fig. 2c) that RANKL was derived from CD4^+^ CD25^+^ T cells in CRC. This is the first time this result has been reported for CRC, and it is consistent with prior findings for other cancers [16]. This evidence suggests that RANKL/RANK and the TME in CRC are closely related.

Previous research has linked RANKL/RANK to the metastasis of CRC [13] and other cancers [16, 28], and it is believed that metastasis is associated with CSCs [32]. Our research demonstrated that RANK could enhance metastasis by promoting the stemness of CRC cells (Fig. 3a, e), consistent with findings in breast cancer [27, 28]. The screening of CSC markers (Fig. S1f) revealed that RANK was able to upregulate stemness markers. In addition, we observed a substantial increase in CD44 levels in CRC cells following RANK overexpression (Fig. 3c). These findings suggest that the RANKL/RANK pathway influences the proliferation, metastasis, and tumorigenesis of CSCs by elevating CD44 levels.

The RANKL/RANK pathway can influence TME composition. The RANKL/RANK pathway has been linked to the release of chemokines that recruit T lymphocytes in breast cancer and endometrial cancer [16, 24]. We discovered that RANKL/RANK can enhance CCL20 production by CRC cells (Fig. 4c), which is consistent with earlier research [24], demonstrating that RANKL/RANK is important in the immunological microenvironment of CRC. Furthermore, CCL20 can influence the TME via immune cells such as B cells, T cells, and dendritic cells, thereby influencing the progression of CRC [33]. Furthermore, Wang et al. demonstrated that CCL20 generated by CRC cells could recruit Tregs to improve chemoresistance [34]. Furthermore, CCR6 expressed on the surface of Tregs as a CCL20 receptor has been investigated in tumor immunity [25, 26, 35]. In this study, we discovered that RANKL/RANK could enhance Tregs recruitment via the CCL20–CCR6 pathway, thereby accelerating the malignant evolution of CRC.

RANK, or receptor activator of nuclear factor kappa-B, activates the NF-κB pathway [36]. The NF-κB pathway is involved in cancer, epithelial–mesenchymal transition, metastasis, chemoresistance, and stemness [34, 36–38]. Nonetheless, knowledge of the NF-κB pathway’s significance in CRC is limited. Indeed, some studies have shown that CRC cells have an active NF-κB pathway; in general, CRC growth is dependent on NF-κB signaling [34]. RANK has been found to activate the NF-κB pathway, promoting the malignant progression of breast cancer [36]. RANK can upregulate CCL20 (Fig. 7a–c) and increase CRC stemness (Figs. 3c, 6b) via the NF-κB pathway, according to our findings. Using the ChIP-QPCR experiment, we also revealed that P65 can bind to the promoter of CCL20 (Fig. 7d, e). Taken together, our findings suggest that the NF-κB pathway is critical in the recruitment of immune cells and the advancement of CRC malignancy.

In summary, we associated Tregs with CRC stemness via RANKL/RANK signaling and discovered a new mechanism by which Tregs enhance CRC metastasis. Namely, Tregs trigger the RANK signaling of CRC cells by secreting RANKL, thereby promoting CRC metastasis by increasing stemness. Activation of the RANKL/RANK signaling pathway can have a positive feedback effect by recruiting more Tregs via the CCL20–CCR6 pathway, thereby controlling CRC metastasis (Fig. 7f). The RANKL/RANK pathway and the TME are known to interact throughout development and cancer, but the underlying mechanism is unknown. Finally, given the role of RANKL/RANK signaling in tumorigenesis, anticancer drug clinical trials in the appropriate molecular targets (such as CCL20/CCR6) and RANKL/RANK inhibitors used in combination, or licensing effectively inhibit CRC metastasis, improve treatment effectiveness. Our findings contribute to a better understanding of the RANKL/RANK signaling pathway as it relates to the immunological microenvironment, stemness, and metastasis of CRC.

### Supplementary information

Supplementary Materials

western blot_raw_images

## Data Availability

The authors declare that all the data supporting the findings in this study are available in this study and are available from the corresponding author through reasonable request.
