# Supplementary material for: RANKL/RANK signaling recruits Tregs via the CCL20–CCR6 pathway and promotes stemness and metastasis in colorectal cancer
Source: Cell Death Dis. 2024 Jun 20;15(6):437. doi: 10.1038/s41419-024-06806-3 (PMC11190233; doi:10.1038/s41419-024-06806-3)
Supplement: Supplementary file 1 — Supplementary Materials [file 41419_2024_6806_MOESM1_ESM.docx]

**Supplementary Figures**


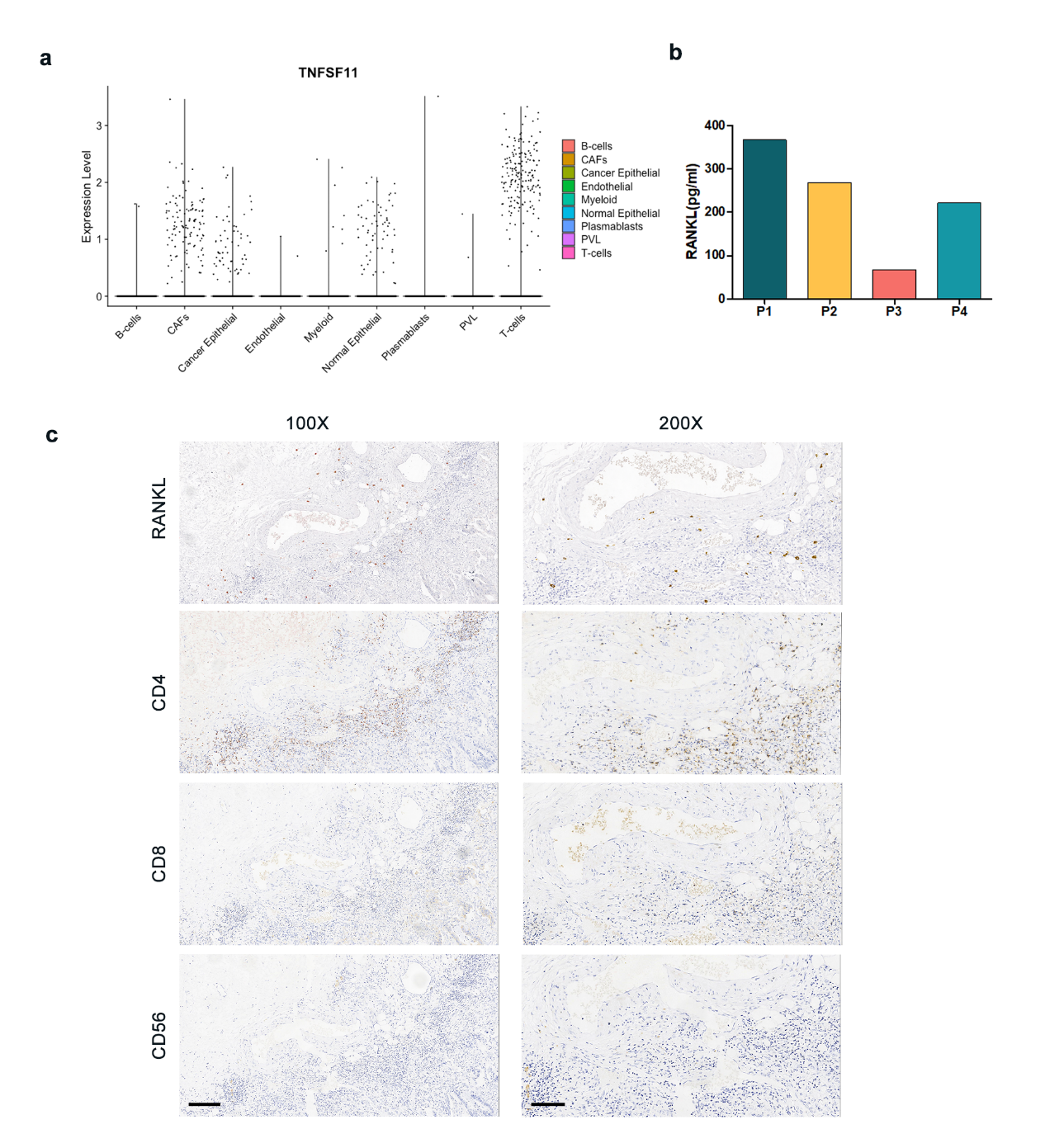


Figure S1. RANKL is mainly derived from CD4+ T cells.

(a) The origin of RANKL(TNFSF11) was analyzed through a single cell database.

(b) ELISA of RANKL protein expressions in CD3^+^ T cells of CRC patients (P1–4: four different samples from CRC patients)

(c) IHC co-stain RANKL with multiple markers of immune cells (CD4, CD8, CD56) in CRC patients. (100×, 200×)

Scales bars = 200 μm (100×), 100 μm (200×), and 50 μm (400×).


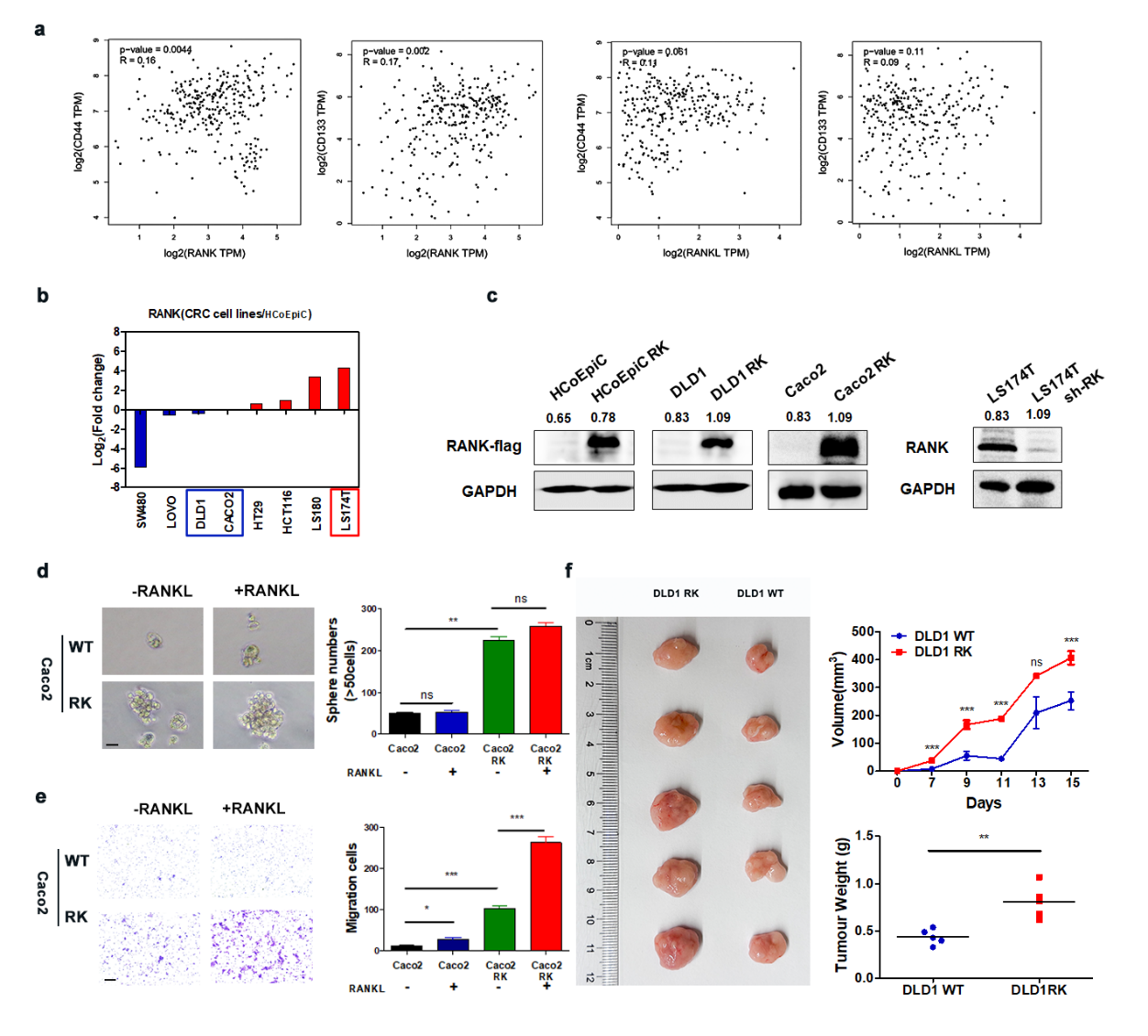


Figure S2. RANK promotes malignant progression of CRC.

(a) The mRNA level correlation between RANK/RANKL and CD44/CD133 in CRC tissues was detected by analyzing the dataset GEPIA.

(b) Log2 fold changes in related RANK mRNA expression of CRC cell lines compared to the normal cell (HCoEpiC).

(c) RANK stable overexpression or knockdown efficiency was confirmed by western blotting in CRC cells.

(d) RANK overexpression influenced the sphere formation (n=3) and (e) the migration (n=5) of Caco2 cells. And the addition of 100 ng/ml RANKL moderately increased the migration of Caco2 cells. (Sphere formation: 400×, Migration: 200×)

(f) The subcutaneous tumors formed by control and DLD1 RK cells were obtained from NOD/Scid mice. Both the volume and weight of subcutaneous tumors were shown in the right panel (mean ± SD, n = 5).

Scales bars = 200 μm (100×), 100 μm (200×), and 50 μm (400×). *P < 0.05, **P < 0.01, ***P < 0.001, ns, no significance.


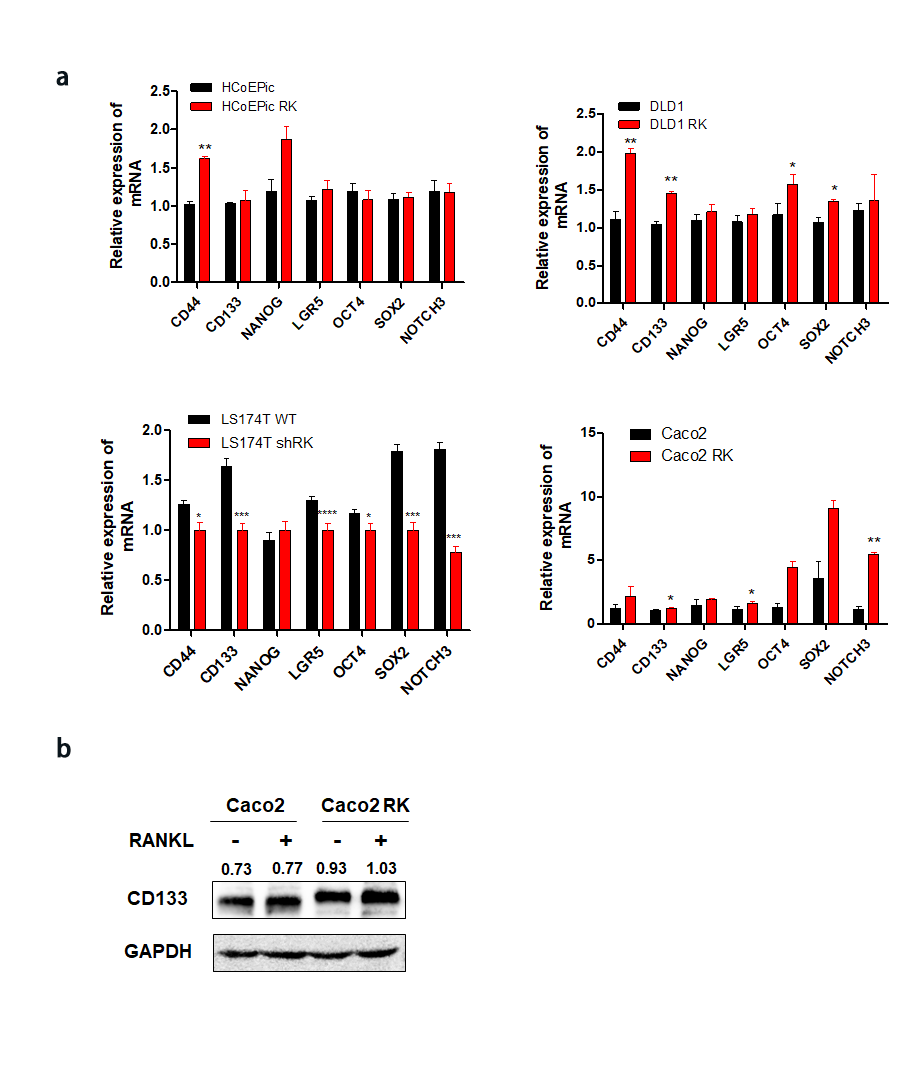


Figure S3. RANK promotes stemness of CRC.

(a) Relative expression of related stemness markers (CD44, CD133, NANOG, LGR5, OCT4, SOX2, NOTCH3) in RANK overexpression or knockdown cells was analyzed by q RT-PCR (n=3).

(b) Western blotting of CD133 protein expressions in RANK overexpression Caco2 cells and the addition of 100 ng/ml RANKL.

*P < 0.05, **P < 0.01, ***P < 0.001.


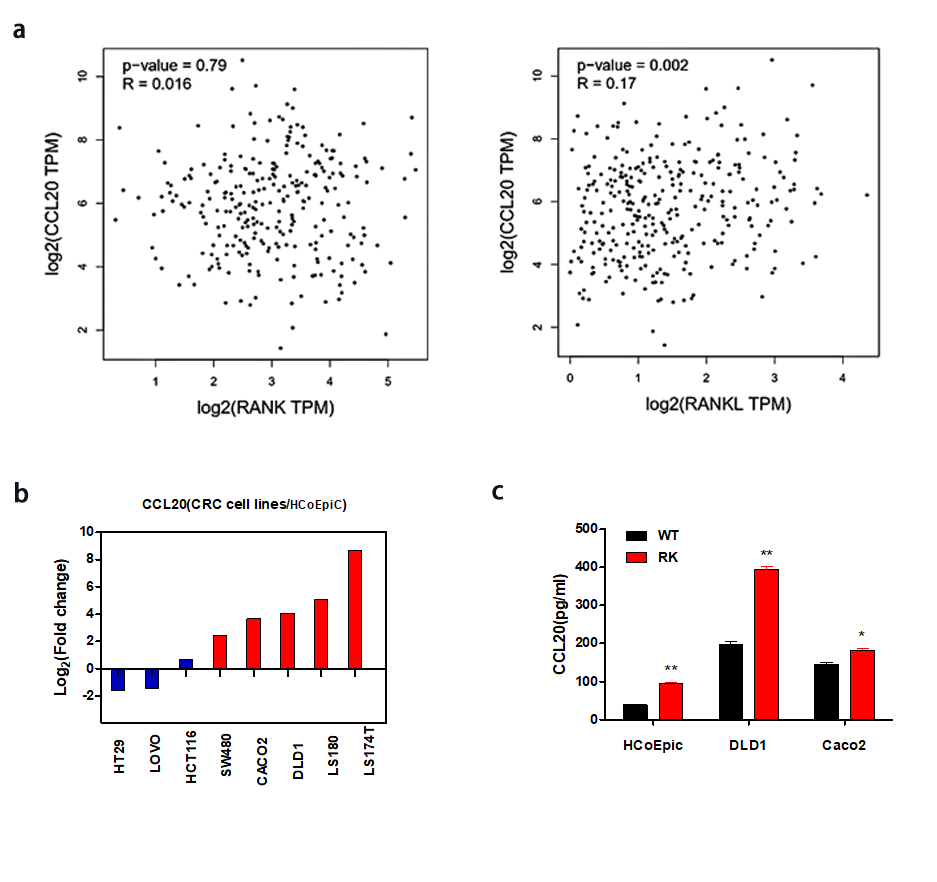


Figure S4. RANK is associated with CCL20 in CRC.

(a) The mRNA level correlation between RANK/RANKL and CCL20 in CRC tissues was detected by analyzing the dataset GEPIA.

(b) Log2 fold changes in related CCL20 mRNA expression of CRC cell lines compared to the normal cell (HCoEpiC) (n=3).

(c)ELISA of CCL20 protein expressions in RANK overexpression cells; error bar indicates mean + SD of 2 technical replicates.

*P < 0.05, **P < 0.01, ***P < 0.001.


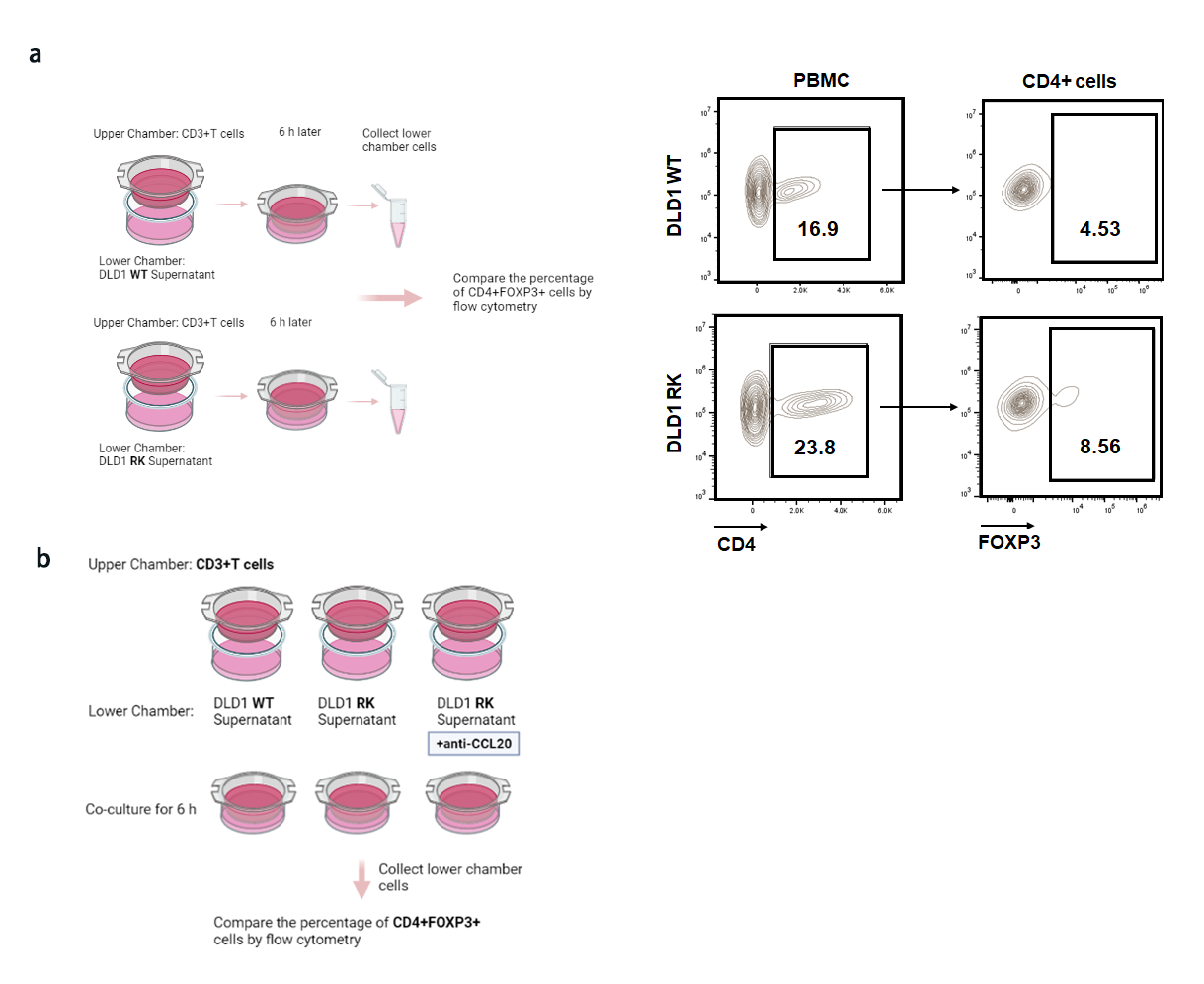


Figure S5. Overexpressed RANK in CRC cells can recruit more CD4^+^FOXP3^+^T cells.

(a) Migration of PBMC from CRC patients co-cultured with the supernatants of DLD1 cells or RANK-overexpressing DLD1 cells was analyzed by transwell assay and flow cytometry, (b) and the addition of anti-CCL20 antibody. (The illustration created with BioRender.com)


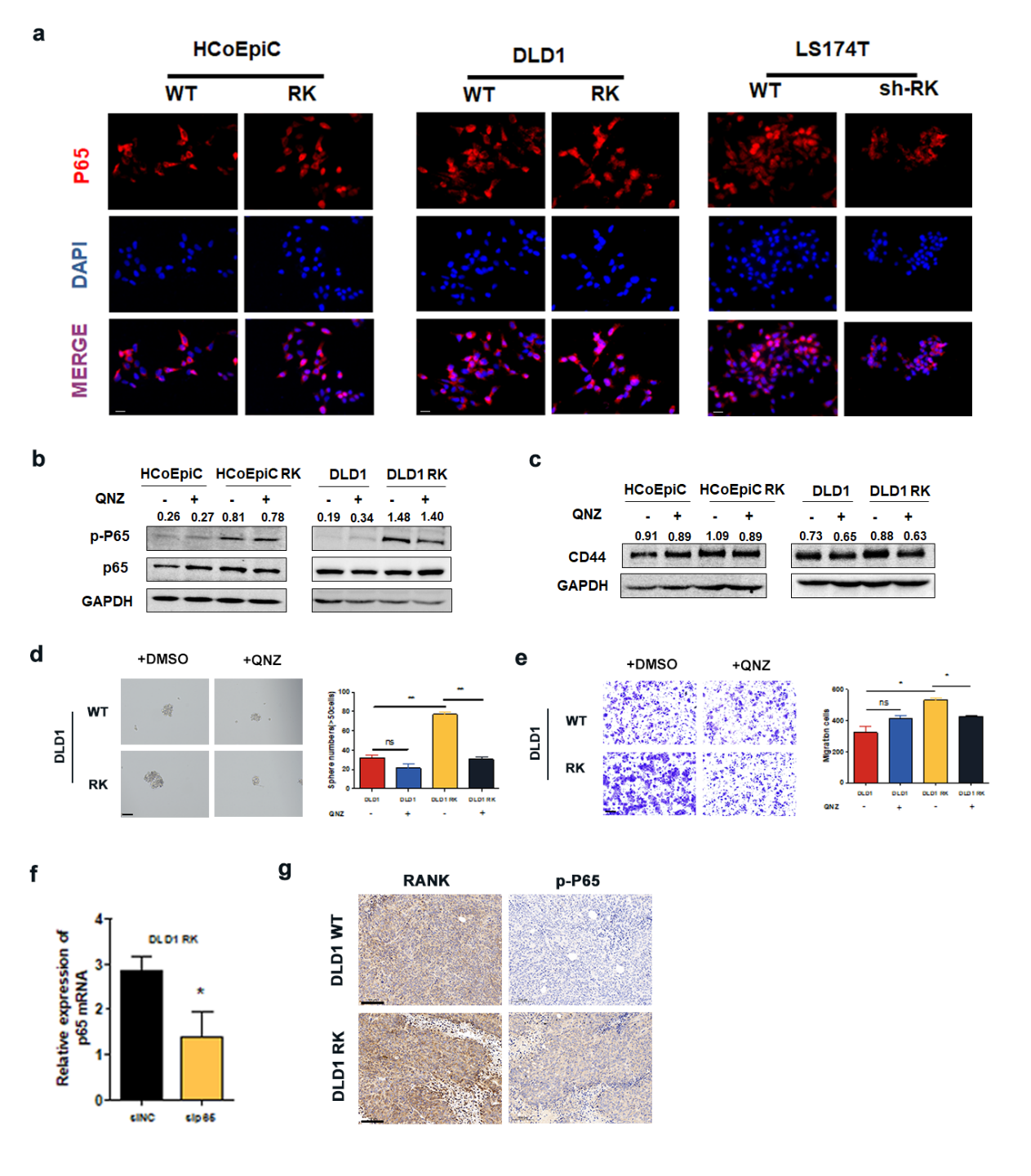


Figure S6. NF-κB inhibitors/ siRNA of P65 can inhibit the stemness of CRC.

(a) Immunofluorescence of P65 protein expressions in RANK overexpression or knockdown cells (400×).

(b) Western blotting of p-P65/P65 protein or (c) CD44 protein expressions in RANK-overexpressing DLD1 cells (DLD1 RK cells) and the addition of 50nm QNZ.

(d) RANK overexpression or addition of 50nm QNZ influenced the sphere formation (n=3) and (e) migration (n=5) of DLD1 cells. (Sphere formation: 400×, Migration: 200×)

(f) Changes of P65 mRNA levels in DLD1 RK cells add P65 siRNA treatment (n=3).

(g) IHC staining was used to detect the expression of RANK and p-P65 in indicated subcutaneous tumors of NOD/Scid mice. (200×)

Scales bars = 200 μm (100×), 100 μm (200×), and 50 μm (400×). *P < 0.05, **P < 0.01, ***P < 0.001, ns, no significance.

**Supplementary Tables**

**Table S1** **Correlation between RANKL expression and clinicopathologic features of 183 CRC patients.**

| Features | N of cases | RANKL | | *p*-value  (χ^2^ tests) |
| --- | --- | --- | --- | --- |
|  |  | Low | High |  |
| Total |  |  |  |  |
| Age (years) |  |  |  |  |
| ＜65 | 109 | 60 | 66 | 0.496 |
| ≥ 65 | 74 | 35 | 39 |  |
| Gender |  |  |  |  |
| Male | 112 | 57 | 55 | 0.023 |
| Female | 71 | 24 | 47 |  |
| CEA level (ng/ml) |  |  |  |  |
| ≤ 5 ng | 61 | 30 | 31 | 0.344 |
| > 5 | 122 | 51 | 71 |  |
| Depth of invasion |  |  |  |  |
| T1 | 3 | 1 | 2 | **0.003** |
| T2 | 28 | 19 | 9 |  |
| T3 | 125 | 56 | 69 |  |
| T4 | 27 | 5 | 22 |  |
| Lymph node metastasis |  |  |  |  |
| N0 | 106 | 61 | 45 | **<0.0001** |
| N1 | 55 | 18 | 37 |  |
| N2 | 22 | 2 | 20 |  |
| Distant metastasis |  |  |  |  |
| M0 | 156 | 78 | 78 | **<0.0001** |
| M1 | 27 | 3 | 24 |  |
| TNM stage (AJCC) |  |  |  |  |
| I | 25 | 16 | 9 | **<0.0001** |
| II | 64 | 43 | 21 |  |
| III | 67 | 19 | 48 |  |
| IV | 27 | 3 | 24 |  |

The bold number represents the *P*-values with significant differences.

**Table S2.** **Target sequences of siRNA**

| Name | Target sequences |
| --- | --- |
| si-RELA | GAGGACATTGAGGTGTATT |
|  |  |

**Table S3.** **Target sequences of shRNA**

| Name | Target sequences |
| --- | --- |
| sh-TNFRSF11A | TGTTTACTTGCCCGGTTTAAT |

**Table S4.** **Sequences of primers**

| Name |  | Sequences |
| --- | --- | --- |
| GAPDH | Forward | 5'-GTCTCCTCTGACTTCAACAGCG-3' |
|  | Reverse | 5'-ACCACCCTGTTGCTGTAGCCAA-3' |
| RANK | Forward | 5'-GCTCAACAAGGACACAGTGTGC-3' |
|  | Reverse | 5'-CGCATCGGATTTCTCTGTCCCA-3' |
| CD44 | Forward | 5'-TGGCACCCGCTATGTCCAG-3' |
|  | Reverse | 5'-GTAGCAGGGATTCTGTCTG-3' |
| CD133 | Forward | 5'-CACTACCAAGGACAAGGCGTTC-3' |
|  | Reverse | 5'-CAACGCCTCTTTGGTCTCCTTG-3' |
| OCT4 | Forward | 5'-CCTGAAGCAGAAGAGGATCACC-3' |
|  | Reverse | 5'-AAAGCGGCAGATGGTCGTTTGG-3' |
| Nanog | Forward | 5'-CTCCAACATCCTGAACCTCAGC-3' |
|  | Reverse | 5'-CGTCACACCATTGCTATTCTTCG-3' |
| Sox2 | Forward | 5'-GCTACAGCATGATGCAGGACCA-3' |
|  | Reverse | 5'-TCTGCGAGCTGGTCATGGAGTT-3' |
| Notch3 | Forward | 5'-TACTGGTAGCCACTGTGAGCAG-3' |
|  | Reverse | 5'-CAGTTATCACCATTGTAGCCAGG-3' |
| LGR5 | Forward | 5’-CCTGCTTGACTTTGAGGAAGACC-3' |
|  | Reverse | 5’-CCAGCCATCAAGCAGGTGTTCA-3’ |
| CCL2  CCL3  CCL11  CCL17  CCL20  CXCL1  CXCL5  CXCL8  CXCL9  CXCL10  CXCL11  RELA | Forward  Reverse  Forward  Reverse  Forward  Reverse  Forward  Reverse  Forward  Reverse  Forward  Reverse  Forward  Reverse  Forward  Reverse  Forward  Reverse  Forward  Reverse  Forward  Reverse  Forward  Reverse | 5’-AGAATCACCAGCAGCAAGTGTCC-3’  5’-TCCTGAACCCACTTCTGCTTGG-3’  5’-ACTTTGAGACGAGCAGCCAGTG-3’  5’-TTTCTGGACCCACTCCTCACTG-3’  5’-GCTACAGGAGAATCACCAGTGG-3’  5’-GGAATCCTGCACCCACTTCTTC-3’  5’-TTCTCTGCAGCACATCCACGCA-3’  5’-CTGGAGCAGTCCTCAGATGTCT-3’  5’-AAGTTGTCTGTGTGCGCAAATCC-3’  5’-CCATTCCAGAAAAGCCACAGTTTT-3’  5’-AGCTTGCCTCAATCCTGCATCC-3’  5’-TCCTTCAGGAACAGCCACCAGT-3’  5’-CAGACCACGCAAGGAGTTCATC-3’  5’-TTCCTTCCCGTTCTTCAGGGAG-3’  5’-GAGAGTGATTGAGAGTGGACCAC-3’  5’-CACAACCCTCTGCACCCAGTTT-3’  5’-CTGTTCCTGCATCAGCACCAAC-3’  5’-TGAACTCCATTCTTCAGTGTAGCA-3’  5’-GGTGAGAAGAGATGTCTGAATCC-3’  5’-GTCCATCCTTGGAAGCACTGCA-3’  5’-AAGGACAACGATGCCTAAATCCC-3’  5’-CAGATGCCCTTTTCCAGGACTTC-3’  5’- TGAACCGAAACTCTGGCAGCTG -3’  5’- CATCAGCTTGCGAAAAGGAGCC -3’ |

Table S5: CSCs frequency calculated by ELDA in Limiting dilution assay.

| Dose（number of cells） | DLD1 WT | | | | DLD1 RK | |
| --- | --- | --- | --- | --- | --- | --- |
|  | Tested | Response | | | Tested | Response |
| 2x10^7^ | 5 | | 3 | 5 | | 5 |
| 1x10^7^ | 5 | | 1 | 5 | | 4 |
| 1x10^6^ | 5 | | 0 | 5 | | 1 |
| Frequency | 1/41742463 | | | | 1/5248042 | |

**Table S6 CCL20expression (tumor vs normal) and clinicopathologic features of 8 CRC patients.**

| Patient  number | CCL20 expression  (tumor vs normal) | Age | Gender | Type | T（Depth of invasion | N（Lymph node metastasis | M（Distant metastasis |
| --- | --- | --- | --- | --- | --- | --- | --- |
| P1 | High*** | 50 | Female | Rectum | 4 | 1 | 1 |
| P2 | High*** | 41 | Female | Rectum | 3 | 2 | 0 |
| P3 | no significance | 74 | Male | Rectum | 1 | 0 | 0 |
| P4 | High** | 57 | Female | Colon | 4 | 1 | 0 |
| P5 | Low*** | 61 | Female | Rectum | 3 | 0 | 0 |
| P6 | High** | 55 | Male | Rectum | 2 | 0 | 0 |
| P7 | Low*** | 83 | Male | Colon | 2 | 0 | 0 |
| P8 | High*** | 60 | Male | Colon | 4 | 0 | 0 |
