# Supplementary figures and images for: RANKL/RANK signaling recruits Tregs via the CCL20–CCR6 pathway and promotes stemness and metastasis in colorectal cancer
Source: Cell Death Dis. 2024 Jun 20;15(6):437. doi: 10.1038/s41419-024-06806-3 (PMC11190233; doi:10.1038/s41419-024-06806-3)

Figure 1_raw_images


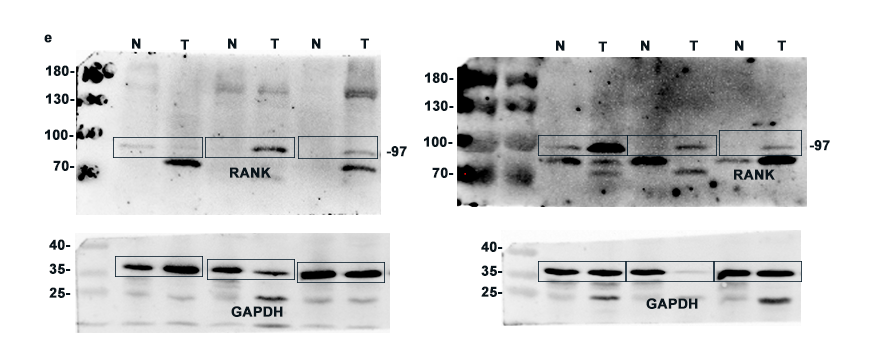


Figure 3_raw_images


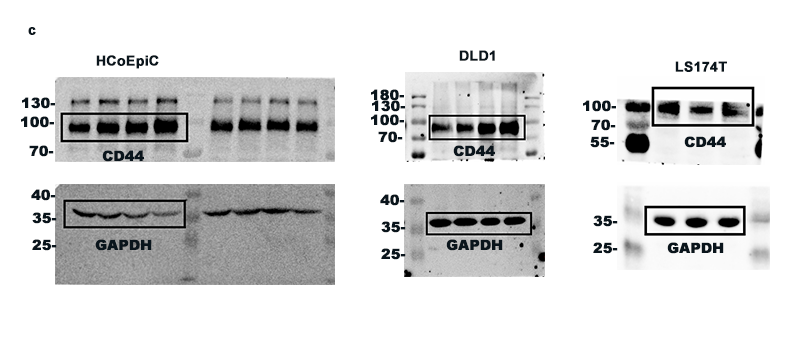


Figure 6_raw_images


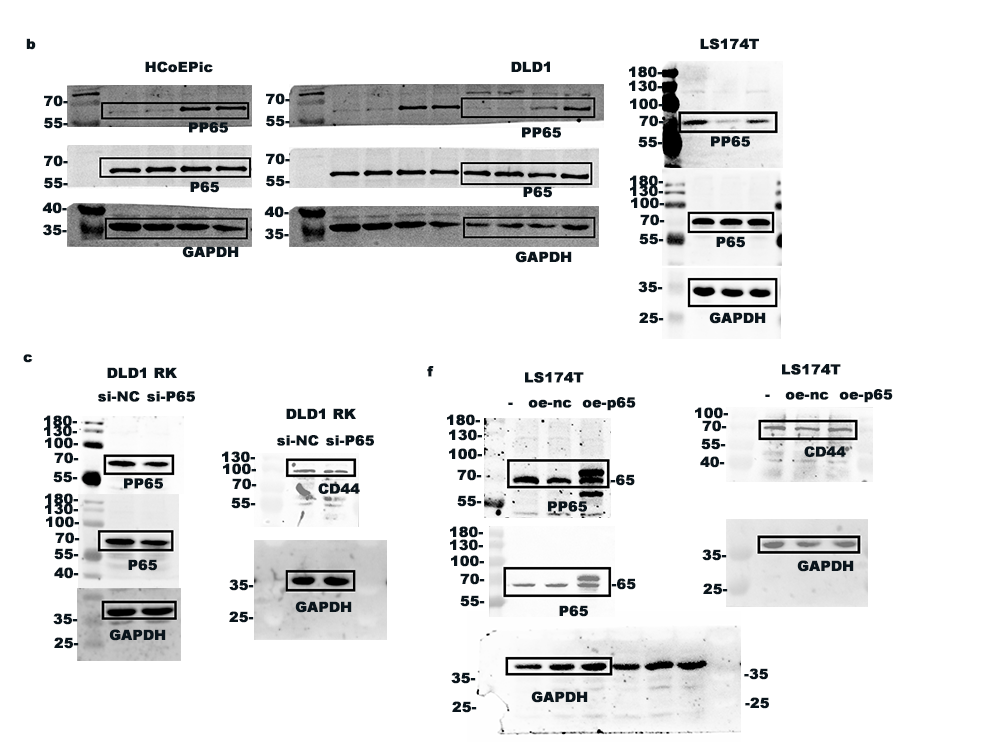


Figure S2_raw_images


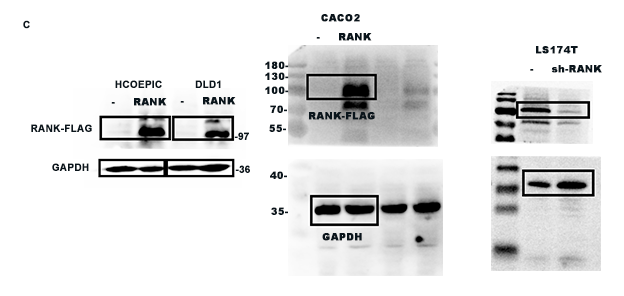


Figure S6_raw_images


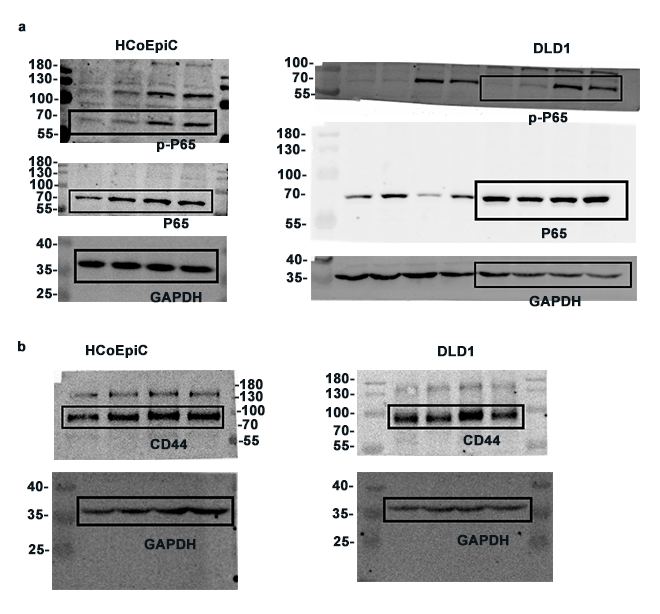

Supplement: Supplementary file 2 — western blot_raw_images [file 41419_2024_6806_MOESM2_ESM.docx]
